# Possible use of repeated cold stress for reducing fatigue in chronic fatigue syndrome: a hypothesis

**DOI:** 10.1186/1744-9081-3-55

**Published:** 2007-10-24

**Authors:** Nikolai A Shevchuk

**Affiliations:** 1 Molecular Radiobiology Section, the Department of Radiation Oncology, Virginia Commonwealth University School of Medicine, 401 College St, Richmond, VA 23298, USA

## Abstract

**Background:**

Physiological fatigue can be defined as a reduction in the force output and/or energy-generating capacity of skeletal muscle after exertion, which may manifest itself as an inability to continue exercise or usual activities at the same intensity. A typical example of a fatigue-related disorder is chronic fatigue syndrome (CFS), a disabling condition of unknown etiology and with uncertain therapeutic options. Recent advances in elucidating pathophysiology of this disorder revealed hypofunction of the hypothalamic-pituitary-adrenal axis and that fatigue in CFS patients appears to be associated with reduced motor neurotransmission in the central nervous system (CNS) and to a smaller extent with increased fatigability of skeletal muscle. There is also some limited evidence that CFS patients may have excessive serotonergic activity in the brain and low opioid tone.

**Presentation of the hypothesis:**

This work hypothesizes that repeated cold stress may reduce fatigue in CFS because brief exposure to cold may transiently reverse some physiological changes associated with this illness. For example, exposure to cold can activate components of the reticular activating system such as raphe nuclei and locus ceruleus, which can result in activation of behavior and increased capacity of the CNS to recruit motoneurons. Cold stress has also been shown to reduce the level of serotonin in most regions of the brain (except brainstem), which would be consistent with reduced fatigue according to animal models of exercise-related fatigue. Finally, exposure to cold increases metabolic rate and transiently activates the hypothalamic-pituitary-adrenal axis as evidenced by a temporary increase in the plasma levels of adrenocorticotropic hormone, beta-endorphin and a modest increase in cortisol. The increased opioid tone and high metabolic rate could diminish fatigue by reducing muscle pain and accelerating recovery of fatigued muscle, respectively.

**Testing the hypothesis:**

To test the hypothesis, a treatment is proposed that consists of adapted cold showers (20 degrees Celsius, 3 minutes, preceded by a 5-minute gradual adaptation to make the procedure more comfortable) used twice daily.

**Implications of the hypothesis:**

If testing supports the proposed hypothesis, this could advance our understanding of the mechanisms of fatigue in CFS.

## Background

There seems to be no universally accepted definition of biological fatigue [1], although it is often defined as a reduction in the force output and/or energy-generating capacity of skeletal muscle after exertion, which may manifest itself as an inability to continue exercise or usual activities at the same intensity [1-4]. Fatigue is thought to be associated with a diminished contractile ability of muscles due to accumulation of lactic acid and depletion of energy stores (glycogen) [5,6] as well as with a reduction in motor neurotransmission delivered to skeletal muscle by the central nervous system (CNS), all of which can result in diminished force output [7,8]. At the level of the CNS, precise mechanisms of fatigue are not well understood, although there is some evidence that it may be associated with diminished activity of a brainstem structure called reticular activating system [9-11] and with increased levels of serotonin in the frontal cortex and hippocampus [12,13].

A typical fatigue-related disorder is chronic fatigue syndrome (CFS), a complex and disabling condition characterized by extended periods of severe fatigue unexplained by known medical causes [14]. Currently, there are no specific diagnostic tests and etiology of CFS remains elusive, although some progress has been made in elucidating its pathophysiology [15,16]. A number of studies have reported insufficient function of the hypothalamic-pituitary-adrenal (HPA) axis (e.g. lowered production of cortisol [16,17]) and a rather frequent occurrence of autonomic nervous system dysfunction in patients with CFS [16,18]. Neither lowered production of cortisol nor dysautonomia appears to be a causative factor in most CFS patients because cortisol injections and various therapeutic approaches to autonomic nervous system abnormalities have so far shown a rather limited effect on CFS symptoms [17,19,20]. Other findings about the pathophysiology of CFS suggest that abnormal fatigability in this disorder is associated with a reduced ability of the CNS to recruit motor neurons [21-23] and with some biochemical abnormalities in skeletal muscle [24-26]. In addition, there is some evidence that CFS patients may have a low opioid tone ([27-30], contrary evidence: [31,32]), as well as an increased level of serotonergic activity in the brain ([33-38], contrary evidence: [39,40]). The latter has been shown to correlate with fatigue in animal models of exercise-related fatigue [13,41-45].

This paper describes a physiological treatment, namely, exposure to moderate cold, which could have a beneficial effect on some of the above-mentioned pathological changes as explained in more detail below.

## Presentation of the hypothesis

It is known that small amounts of stressful or harmful agents can be beneficial for animals, a phenomenon known as hormesis [46,47]. In particular, exposure to cold can transiently reverse several physiological changes that are often associated with CFS and therefore, the hypothesis is that repeated cold stress can reduce fatigue in CFS patients. The following is detailed theoretical evidence that appears to support this hypothesis.

1) Insufficient function of the HPA axis has been found to correlate with fatigue [48], for example, a lowered plasma level of stress hormone cortisol (secreted by adrenal glands) is one of the few consistent endocrine changes found in CFS in numerous studies [17], although the level of cortisol in CFS patients is within the normal range and therefore cannot be used as a diagnostic tool [16]. Cold stress is known to transiently activate the HPA axis [49,50] as evidenced by a brief increase in the plasma levels of adrenocorticotropic hormone [51,52] and beta-endorphin (the latter is a secreted by the pituitary gland) [53,54], as well as a modest elevation in the level of cortisol [55,56]. Some studies reported no significant change in cortisol levels following cold stress [57,58], which may be due to gender and diurnal variation of this effect [55,56]. In addition, there is some evidence of another deficiency of the HPA axis in various disorders associated with fatigue: hypofunction of corticotropin-releasing hormone-producing neurons (located in hypothalamus) [48,59,60]. Therefore, "exercising" the HPA system by repeated exposure to cold could potentially restore its normal function in CFS, or at least increase the net HPA activity (without a change in baseline activity [61]) and, possibly, reduce fatigue. For example, repeated cold stress has been shown to enhance HPA axis responsiveness to other stressors [62,63] and to enhance cortisol responses to cold stress [64].

2) Cold hydrotherapy is known to produce a significant analgesic effect [65-67], which could be beneficial in CFS, where pain symptoms are rather common [68,69]. The cold stress-induced analgesia is believed to be mediated by increased production of opioid peptide beta-endorphin, which is an endogenous pain-killer [53,55,70,71].

3) Exposure to cold is known to increase metabolic rate: for instance, head-out immersion in cold water of 20°C almost doubles metabolic rate, while at 14°C it is more than quadrupled [72]. Theoretically, the high metabolic rate may accelerate [73,74] the process of recovery of muscle tissues from fatigue in CFS [24,25,75,76] and some studies indeed show accelerated muscle recovery following immersion in cold water [77,78]. In combination with cold-induced analgesia described above, the increased metabolic rate would be expected to reduce fatigue by both improving muscle recovery after exertion and by reducing muscle pain [79]. A cold-induced increase in cerebral metabolic rate [80] may also be consistent with reduced fatigue ([7,81] contrary evidence: [82,83]).

4) There is evidence that exposure to cold can activate some components of the brainstem arousal system [84-86] (also known as the reticular activating system [87,88]). In particular, cold stress appears to stimulate activity of serotonergic neurons of raphe nuclei [84,89-91] and noradrenergic neurons of locus ceruleus [84,92], the situation that would be consistent with activation of behavior and enhanced somatomotor function of the brain [9,87,93-96]. This could be beneficial in CFS because abnormally high fatigability of CFS patients appears to be mediated by a reduction in the ability of the CNS to generate motor neurotransmission [21-23]. It is noteworthy that in polio survivors and patients with multiple sclerosis, the presence of minor lesions in the reticular activating system correlates with severe chronic fatigue [10,97]. This kind of lesions can also cause lethargy in laboratory animals [9,88,98]. Reduced electrical activity in the reticular activating system also appears to correlate with fatigue in laboratory animals [99-101]. At present, there is no evidence that CFS patients have lesions in the reticular activating system [9,102], although there is some limited evidence of abnormalities of metabolism, blood flow, and electrical activity in the brainstem [81,103-105], the anatomical site of the reticular activating system [87,88].

5) While the increased level of serotonin in the brainstem [85] is thought to correlate with arousal and increased cortical activity [11,94,106], high levels of serotonin in other areas of the brain, particularly in the hippocampus and frontal cortex, are believed to be associated with fatigue, which is the basis of "the serotonin hypothesis of central fatigue" [12,13,41-45]. Whether high levels of brain serotonin actually cause fatigue or are merely an epiphenomenon is a subject of controversy [12,13]. With respect to cold stress, studies suggest that it reduces the level of serotonin in most regions of the brain [107,108] except the rostral brainstem [85], which would be consistent with diminished fatigue [12,13] and could be beneficial in CFS ([33-38], contrary evidence: [39,40]).

6) Exposure to cold typically causes activation of the sympathetic nervous system (SNS) [49,109], which, theoretically, can be undesirable in CFS because there is evidence of hyperactivity of some components of the SNS in CFS patients [18,110]. It should be noted that physical exercise is also known to transiently activate the SNS [111] and graded exercise appears to be beneficial in CFS [112,113]. Therefore, brief cold stress will not necessarily have adverse effects on CFS patients (more detailed discussion can be found in Additional file 1).

7) As described previously, brief cold hydrotherapy appears to be safe and does not seem to have either short-term or long-term adverse effects on health [109,114-117]. The effect of moderately cold hydrotherapy (16–23°C) on core body temperature is expected to be very small and therefore hypothermia is hardly a concern [118-120].

## Testing the hypothesis

To test the hypothesis, a treatment is proposed that consists of adapted cold showers (20°C, 3 minutes, preceded by 5-minute gradual adaptation) twice a day. The detailed study protocol can be found in Additional file 2. Statistically insignificant preliminary evidence is described in Additional file 3.

## Implications of the hypothesis

If statistically significant studies confirm (or refute) the hypothesis, this could further our understanding of the mechanisms of physiological fatigue and possibly contribute to the development of new therapeutic approaches to CFS.

## Competing interests

The author(s) declare that they have no competing interests.

## Authors' contributions

The idea and writing are solely N.A.S.'s.

## Supplementary Material

Additional file 1 **Sympathetic nervous system and chronic fatigue syndrome**. The file name is Additional_File_1.pdf and it contains a brief review of literature on the sympathetic nervous system abnormalities observed in patients with chronic fatigue syndrome. The file contains its own list of references separate from the main text. Click here for file

Additional file 2 **Proposed study design**. The file name is Additional_File_2.pdf and it contains a detailed protocol of the proposed study including statistical estimates of the sample size. The file contains its own list of references separate from the main text. Click here for file

Additional file 3 **Limited preliminary evidence**. The file name is Additional_File_3.pdf and it provides a detailed description of preliminary evidence that appears to support the hypothesis. The file contains its own list of references separate from the main text. Click here for file
