# Supplementary material for: Possible use of repeated cold stress for reducing fatigue in chronic fatigue syndrome: a hypothesis
Source: Behav Brain Funct. 2007 Oct 24;3:55. doi: 10.1186/1744-9081-3-55 (PMC2164952; doi:10.1186/1744-9081-3-55)
Supplement: Additional file 1 — Sympathetic nervous system and chronic fatigue syndrome. The file name is Additional_File_1.pdf and it contains a brief review of literature on the sympathetic nervous system abnormalities observed in patients with chronic fatigue syndrome. The file contains its own list of references separate from the main text. [file 1744-9081-3-55-S1.pdf]

Additional file 1

Sympathetic nervous system and chronic fatigue syndrome

In literature on chronic fatigue syndrome (CFS), there seems to be no consensus on whether this disorder is characterized by hyperfunction [121], normalcy [122] or hypofunction [123] of the sympathetic nervous system (SNS), although it is generally accepted that CFS patients tend to have some kind of dysfunction of the autonomic nervous system [124,125]. This dysautonomia may present as a combination of abnormally high and low test values obtained from various assays of the SNS and parasympathetic nervous system functions [125,126]. It is known that increased activity of the SNS typically results in vasoconstriction in most tissues (except for brain parenchyma [127] and skeletal muscle), elevated blood pressure, increased heart rate, and an elevated plasma level of norepinephrine (one of the major sympathetic neurotransmitters) [128-130]. Reduced activity of the SNS will usually have the opposite effects [131]. Below is a brief overview of literature on the status of these physiological variables in CFS:

a) Estimates of baseline vascular tone in CFS patients, as measured by total peripheral resistance (mean arterial pressure divided by cardiac output), suggest that there may be a trend toward greater peripheral vasoconstriction associated with CFS [132-134]. On the other hand, the change of peripheral vascular resistance in CFS patients in response to a head up tilt challenge appears to be within the normal range [135].

b) Although many studies report that CFS is often associated with a decrease in blood pressure during either an orthostatic or tilt challenge [124,126,136-139] there are reports of increased blood pressure in CFS patients under somewhat different experimental conditions of head up tilt [133,140]. Baseline blood pressure in CFS patients is within the

normal range according to some studies [139,140] and may be elevated according to others [132].

c) Many studies report development of tachycardia in a significant percentage of CFS patients during an orthostatic or tilt challenge [126,137,139,141]. Baseline heart rate in CFS is within the normal range according to some studies [139,140] and elevated according to others [132,135,142].

d) The baseline level of norepinephrine in plasma appears to be normal [122,143,144], although some studies show elevated levels of norepinephrine in CFS patients [145]. Some studies report exaggerated responses of plasma norepinephrine to standing for 10 minutes [137]. On the other hand, the response of plasma norepinephrine to exercise appears to be within the normal range in CFS [122].

The average age and gender differences among the reports cited above make it rather difficult to compare them directly. Nevertheless, this brief and superficial overview suggests that studies of plasma norepinephrine, heart rate, and peripheral resistance point to either normal or excessive activity of the SNS in CFS patients. On the other hand, data from studies on blood pressure can be interpreted as hyperfunction, normalcy or hypofunction of the SNS in patients with CFS. At present, it is not clear if the SNS disturbances are a causative factor of CFS or a consequence of abnormally low physical activity or some other pathology [124,132,142,146]. Further research would be needed to identify a cause of the observed dysautonomia in CFS.

As mentioned in the text, exposure to cold is known to activate the SNS [130,147] and, depending on experimental conditions, this may result in different cardiovascular responses [148]. Cardiovascular effects of 20°C showers, which are proposed in this paper, have never

been studied (at least cannot be found in PubMed). Somewhat paradoxically, head-out immersion of humans in cold water at 20°C *lowers* both blood pressure and heart rate [148] despite significant peripheral vasoconstriction and a more than 4-fold increase in plasma norepinephrine [149]. Since immersion in thermoneutral water (32-34°C) has an almost identical inhibitory effect on blood pressure and heart rate [148] (while it does not increase peripheral vasoconstriction and plasma norepinephrine [150]), it is possible that the cardiovascular response to 20°C water immersion is due to immersion in water rather than exposure to cold. Therefore, cold showers at 20°C may not have a detectable effect on blood pressure and heart rate. Alternatively, a 20°C cold shower may cause an increase in blood pressure and a decrease in heart rate as observed in experiments with exposure to cold air [151]. It should be noted that immersion in colder water (at 14°C) increases both heart rate and blood pressure [130,148]. Systemic exposure to cold (different methods) has not been reported to cause a detectable change in the plasma level of epinephrine [130,152,153]. Finally, repeated exposure to cold does not appear change the baseline level of norepinephrine in healthy test subjects [130].

In conclusion, it is not clear if physiological stimuli that activate the SNS (such as exposure to cold) will have a net beneficial or a net adverse effect on patients with CFS. As mentioned in argument #6, graded exercise appears to benefit CFS patients [154,155] despite transient activation of the SNS during exercise [156]. Therefore, daily brief exposure to cold may or may not aggravate the autonomic nervous system abnormalities that are often observed in CFS patients.

References

121. Wyller VB, Thaulow E, Amlie JP: **Treatment of chronic fatigue and orthostatic intolerance with propranolol.** *J Pediatr* 2007, **150:**654-655.

122. Ottenweller JE, Sisto SA, McCarty RC, Natelson BH: **Hormonal responses to exercise in chronic fatigue syndrome.** *Neuropsychobiology* 2001, **43:**34-41.

123. Neeck G, Crofford LJ: **Neuroendocrine perturbations in fibromyalgia and chronic fatigue syndrome.** *Rheum Dis Clin North Am* 2000, **26:**989-1002.

124. **Chronic fatigue syndrome: possible causes** [http://www.cdc.gov/cfs/cfscauses.htm]

125. Newton JL, Okonkwo O, Sutcliffe K, Seth A, Shin J, Jones DE: **Symptoms of autonomic dysfunction in chronic fatigue syndrome.** *QJM* 2007, **100:**519-526.

126. Freeman R, Komaroff AL: **Does the chronic fatigue syndrome involve the autonomic nervous system?** *Am J Med* 1997, **102:**357-364.

127. Handa Y, Caner H, Hayashi M, Tamamaki N, Nojyo Y: **The distribution pattern of the sympathetic nerve fibers to the cerebral arterial system in rat as revealed by anterograde labeling with WGA-HRP.** *Exp Brain Res* 1990, **82:**493-498.

128. Sinski M, Lewandowski J, Abramczyk P, Narkiewicz K, Gaciong Z: **Why study sympathetic nervous system?** *J Physiol Pharmacol* 2006, **57 Suppl 11:**79-92.

129. Jansen AS, Nguyen XV, Karpitskiy V, Mettenleiter TC, Loewy AD: **Central command neurons of the sympathetic nervous system: basis of the fight-or-flight response.** *Science* 1995, **270:**644-646.

130. Jansky L, Sramek P, Savlikova J, Ulicny B, Janakova H, Horky K: **Change in sympathetic activity, cardiovascular functions and plasma hormone concentrations due to cold water immersion in men.** *Eur J Appl Physiol Occup Physiol* 1996, **74:**148-152.

131. Hayashi T, Shibamoto T, Yamaguchi Y, Wang HG, Tanaka S: **Suppression of sympathetic nervous system is involved in hypotension and bradycardia during hemofiltration in anesthetized dogs.** *Can J Physiol Pharmacol* 1995, **73:**1495-1501.

132. Farquhar WB, Hunt BE, Taylor JA, Darling SE, Freeman R: **Blood volume and its relation to peak O_2 consumption and physical activity in patients with chronic fatigue.** *Am J Physiol Heart Circ Physiol* 2002, **282:**H66-71.

133. Wyller VB, Due R, Saul JP, Amlie JP, Thaulow E: **Usefulness of an abnormal cardiovascular response during low-grade head-up tilt-test for discriminating adolescents with chronic fatigue from healthy controls.** *Am J Cardiol* 2007, **99:**997-1001.

134. Peckerman A, LaManca JJ, Dahl KA, Chemitiganti R, Qureishi B, Natelson BH: **Abnormal impedance cardiography predicts symptom severity in chronic fatigue syndrome.** *Am J Med Sci* 2003, **326:**55-60.

135. Timmers HJ, Wieling W, Soetekouw PM, Bleijenberg G, Van Der Meer JW, Lenders JW: **Hemodynamic and neurohumoral responses to head-up tilt in patients with chronic fatigue syndrome.** *Clin Auton Res* 2002, **12:**273-280.

136. Bou-Holaigah I, Rowe PC, Kan J, Calkins H: **The relationship between neurally mediated hypotension and the chronic fatigue syndrome.** *JAMA* 1995, **274:**961-967.

137. Streeten DH, Thomas D, Bell DS: **The roles of orthostatic hypotension, orthostatic tachycardia, and subnormal erythrocyte volume in the pathogenesis of the chronic fatigue syndrome.** *Am J Med Sci* 2000, **320:**1-8.

138. Cho HJ, Skowera A, Cleare A, Wessely S: **Chronic fatigue syndrome: an update focusing on phenomenology and pathophysiology.** *Curr Opin Psychiatry* 2006, **19:**67-73.

139. Naschitz JE, Rosner I, Rozenbaum M, Gaitini L, Bistritzki I, Zuckerman E, Sabo E, Yeshurun D: **The capnography head-up tilt test for evaluation of chronic fatigue syndrome.** *Semin Arthritis Rheum* 2000, **30:**79-86.

140. Natelson BH, Intriligator R, Cherniack NS, Chandler HK, Stewart JM: **Hypocapnia is a biological marker for orthostatic intolerance in some patients with chronic fatigue syndrome.** *Dyn Med* 2007, **6:**2.

141. Stewart JM, Gewitz MH, Weldon A, Arlievsky N, Li K, Munoz J: **Orthostatic intolerance in adolescent chronic fatigue syndrome.** *Pediatrics* 1999, **103:**116-121.

142. Stewart JM: **Autonomic nervous system dysfunction in adolescents with postural orthostatic tachycardia syndrome and chronic fatigue syndrome is characterized by attenuated vagal baroreflex and potentiated sympathetic vasomotion.** *Pediatr Res* 2000, **48:**218-226.

143. Kavelaars A, Kuis W, Knook L, Sinnema G, Heijnen CJ: **Disturbed neuroendocrine-immune interactions in chronic fatigue syndrome.** *J Clin Endocrinol Metab* 2000, **85:**692-696.

144. Peterson PK, Pheley A, Schroeppel J, Schenck C, Marshall P, Kind A, Haugland JM, Lambrecht LJ, Swan S, Goldsmith S: **A preliminary placebo-controlled crossover trial of fludrocortisone for chronic fatigue syndrome.** *Arch Intern Med* 1998, **158:**908-914.

145. Wyller VB, Godang K, Morkrid L, Saul JP, Thaulow E, Walloe L: **Abnormal thermoregulatory responses in adolescents with chronic fatigue syndrome: relation to clinical symptoms.** *Pediatrics* 2007, **120:**e129-137.

146. Bazelmans E, Bleijenberg G, Van Der Meer JW, Folgering H: **Is physical deconditioning a perpetuating factor in chronic fatigue syndrome? A controlled study on maximal exercise performance and relations with fatigue, impairment and physical activity.** *Psychol Med* 2001, **31:**107-114.

147. Nakamoto M: **Responses of sympathetic nervous system to cold exposure in vibration syndrome subjects and age-matched healthy controls.** *Int Arch Occup Environ Health* 1990, **62:**177-181.

148. Sramek P, Simeckova M, Jansky L, Savlikova J, Vybiral S: **Human physiological responses to immersion into water of different temperatures.** *Eur J Appl Physiol* 2000, **81:**436-442.

149. Castellani JW, Young AJ, Kain JE, Sawka MN: **Thermoregulatory responses to cold water at different times of day.** *J Appl Physiol* 1999, **87:**243-246.

150. Gabrielsen A, Sorensen VB, Pump B, Galatius S, Videbaek R, Bie P, Warberg J, Christensen NJ, Wroblewski H, Kastrup J, Norsk P: **Cardiovascular and neuroendocrine responses to water immersion in compensated heart failure.** *Am J Physiol Heart Circ Physiol* 2000, **279:**H1931-1940.

151. Korhonen I: **Blood pressure and heart rate responses in men exposed to arm and leg cold pressor tests and whole-body cold exposure.** *Int J Circumpolar Health* 2006, **65:**178-184.

152. Marino F, Sockler JM, Fry JM: **Thermoregulatory, metabolic and sympathoadrenal responses to repeated brief exposure to cold.** *Scand J Clin Lab Invest* 1998, **58:**537-545.

153. Picotti GB, Carruba MO, Ravazzani C, Cesura AM, Galva MD, Da Prada M: **Plasma catecholamines in rats exposed to cold: effects of ganglionic and adrenoreceptor blockade.** *Eur J Pharmacol* 1981, **69:**321-329.

154. Moss-Morris R, Sharon C, Tobin R, Baldi JC: **A randomized controlled graded exercise trial for chronic fatigue syndrome: outcomes and mechanisms of change.** *J Health Psychol* 2005, **10:**245-259.

155. Wallman KE, Morton AR, Goodman C, Grove R, Guilfoyle AM: **Randomised controlled trial of graded exercise in chronic fatigue syndrome.** *Med J Aust* 2004, **180:**444-448.

156. Toth MJ, Gardner AW, Arciero PJ, Calles-Escandon J, Poehlman ET: **Gender differences in fat oxidation and sympathetic nervous system activity at rest and during submaximal exercise in older individuals.** *Clin Sci (Lond)* 1998, **95:**59-66.
