# Supplementary material for: Possible use of repeated cold stress for reducing fatigue in chronic fatigue syndrome: a hypothesis
Source: Behav Brain Funct. 2007 Oct 24;3:55. doi: 10.1186/1744-9081-3-55 (PMC2164952; doi:10.1186/1744-9081-3-55)
Supplement: Additional file 2 — Proposed study design. The file name is Additional_File_2.pdf and it contains a detailed protocol of the proposed study including statistical estimates of the sample size. The file contains its own list of references separate from the main text. [file 1744-9081-3-55-S2.pdf]

Additional file 2

Proposed study design

To test the hypothesis, a treatment is proposed that consists of adapted cold showers, 20°C, at a constant flow rate selected from the range 16 to 24 L/min, 3 minutes, preceded by a 5-minute gradual adaptation (expansion of the area of contact with cold water from the feet up, to make the procedure more comfortable), the whole procedure being repeated 2 times per day (morning and afternoon, no later than 7 p.m.).

58 patients with CFS diagnosed according to 1994 Centers for Disease Control criteria [157] would be needed to test if the proposed regimen can affect fatigue scores obtained by means of questionnaires [158-160] as well as other variables. The design of the proposed pilot study would be AB/BA cross-over with wait-list controls [161-163]. The wait-list control groups will consist of patients who receive no anti-fatigue treatments. The patients would be distributed randomly into 2 groups (29 patients each): AB or BA. Group AB will undergo twice daily adapted cold showers for 8 weeks, followed by 2 weeks of wash-out (no treatment), and then 8 weeks of control period (no treatment). The order is reverse for group BA: no treatment for 8 weeks, followed by 2 weeks of wash-out and then 8 weeks of cold hydrotherapy. The total duration of the study is 18 weeks. The fatigue questionnaires would be administered weekly, including immediately before initiation of the study. Additionally, urinary free cortisol (24-hour level) [164] and both baseline and cold-stress response measurements of plasma norepinephrine [165], heart rate and blood pressure [166], tilt table testing [167-170], and central activation failure assay [171] would be performed once a week. To compare these additional variables to those of normal subjects,

58 healthy volunteers (gender- and age-matched) would be recruited to an identical AB/BA crossover study and distributed randomly into 2 groups of 29 as described above for CFS patients. Fatigue questionnaires can be optionally administered to healthy volunteers as well and compared between treatment and control groups of the normal test subjects.

Sample size estimates for each of the above-mentioned variables are summarized in Table 1. All estimates are based on the expectation that the treatment will have an effect size (Cohen's *d*) of 0.5, which corresponds approximately to a change by one half of standard deviation (the after-treatment mean value of a variable in patients is 0.5 standard deviations away from the pre-treatment mean) [172]. This effect size is considered to be moderate [172]. Sample size estimates corresponding to Cohen's $d = 1$ are also shown (Table 2), and although this is usually considered a large effect size [172], it may not always translate into a significant clinical change [176] and therefore may also be considered if the planned study does not include all of the proposed variables.

While the smaller sample size is an attractive advantage of the cross-over design, the possibility of carry-over effects is a potential drawback [161-163]. The proposed 2-week wash-out period is expected to preclude detectable carry-over effects, but if the cross-over design is still unacceptable for some reason [173,174], a parallel design may be considered instead. The parallel trial would last a total of 8 weeks and include 4 groups: two groups of CFS patients, 77 each, and two groups of healthy volunteers, 77 each, according to Table 1; fewer participants if estimates from Table 2 are used and the study does not include all of the proposed variables. Weekly questionnaires and physiological measurements would be administered as described above for the cross-over trial. A "warm shower control", i.e. thermoneutral showers (34°C) performed in a manner similar to the adapted cold showers, may also be considered.

Table 1. Sample size estimates for variables included in the proposed study protocol (Cohen's *d* = 0.5).

The analysis was performed using PASS software [175]; t-test was two-tailed, the probability of Type I error, α, was 5%, and the probability of Type II error, β, was 20% in all calculations. Standard deviation (S.D.) estimates for predicted averages and for differences were calculated as described in [176]. "Group size" means the size of an experimental or control group (ideally, they would be equal). The total number of test subjects in a trial will equal group size times four (two groups of patients plus two groups of healthy volunteers). MFI-20 is Multidimensional Fatigue Inventory [158].

**Variables (and references)**	**Minimal group size**		**Expected effect size (and clinical change [176])**
	**AB/BA cross-over design**	**Parallel design**	
MFI-20 General Fatigue Scale [158,177,178]	23	73	Cohen's *d* = 0.5 (mean is 3.6 S.D. away from normal mean)
MFI-20 Physical Fatigue Scale [158,177,178]	20	56	Cohen's *d* = 0.5 (mean is 2.6 S.D. away from normal mean)
MFI-20 Mental Fatigue Scale [158,177,178]	13	55	Cohen's *d* = 0.5 (mean is 2.1 S.D. away from normal mean)
24-hour urinary free cortisol[1] [164]	27	**77**	Cohen's *d* = 0.5 (mean is 0.2 S.D. away from normal mean)
Change in systolic blood pressure in tilt testing[2] [167]	26	64	Cohen's *d* = 0.5 (mean is 5.8 S.D. away from normal mean)
Central activation failure [171]	24	50	Cohen's *d* = 0.5 (mean is 12.1 S.D. away from normal mean)
Norepinephrine, plasma level[3] [179]	25	59	Cohen's *d* = 0.5 (change in the response to cold)
Heart rate[3] [167,180]	27	64	Cohen's *d* = 0.5 (cold stress-induced change)
Systolic blood pressure[3] [167,180]	**29**	71	Cohen's *d* = 0.5 (cold stress-induced change)

[1] The normal mean and CFS mean are less than 1 S.D. apart.

[2] These data vary widely in literature (Additional file 1); only one report (not necessarily the most representative) was selected randomly for these calculations.

[3] It is not known if these variables will differ significantly between patients and normals (Additional file 1).

Table 2. Sample size estimates with the expected effect size (Cohen's *d*) equal to unity.

The analysis was performed as described in the caption of Table 1. Some of the variables have a modest expected effect size judging by the clinical change (clinical change may be considered insignificant if the mean of the treatment group is 2 standard deviations or farther away from the mean of the normal population [176]). If the planned study only includes variables with a modest expected clinical change, then the sample size can be substantially reduced compared to estimates from Table 1. "Group size" means the size of an experimental or control group (ideally, they would be equal). The total number of test subjects in a trial will equal group size times four (two groups of patients plus two groups of healthy volunteers). MFI-20 is Multidimensional Fatigue Inventory [158].

**Variables (and references)**	**Minimal group size**		**Expected effect size (and clinical change [176])**
	**AB/BA cross-over design**	**Parallel design**	
MFI-20 General Fatigue Scale [158,177,178]	7	19	**Cohen's *d* = 1** (mean is 3.2 S.D. away from normal mean)[1]
MFI-20 Physical Fatigue Scale [158,177,178]	6	15	**Cohen's *d* = 1** (mean is 1.9 S.D. away from normal mean)
MFI-20 Mental Fatigue Scale [158,177,178]	5	15	**Cohen's *d* = 1** (mean is 1.3 S.D. away from normal mean)
24-hour urinary free cortisol[2] [164]	8	20	**Cohen's *d* = 1** (mean is 0.2 S.D. away from normal mean)
Change in systolic blood pressure in tilt testing[3] [167]	8	17	**Cohen's *d* = 1** (mean is 5.3 S.D. away from normal mean)
Central activation failure [171]	7	14	**Cohen's *d* = 1** (mean is 10.2 S.D. away from normal mean)
Norepinephrine, plasma level[4] [179]	11	25	**Cohen's *d* = 1** (change in the response to cold)
Heart rate[4] [167,180]	5	11	**Cohen's *d* = 1** (cold stress-induced change)
Systolic blood pressure[4] [167,180]	6	13	**Cohen's *d* = 1** (cold stress-induced change)

[1] A large value of effect size as expressed by Cohen's *d* greater than 0.8 may not always produce a significant clinical change and vice versa [176]. Different variables listed in this table have a great variability of the ratio of S.D. to the difference between the mean of the (healthy) population and the mean of CFS patients.

[2] The normal mean and CFS mean are less than 1 S.D. apart.

[3] These data vary widely in literature (Additional file 1); only one report (not necessarily the most representative) was selected randomly for these calculations.

[4] It is not known if these variables will differ significantly between patients and normals (Additional file 1).

References

157. Fukuda K, Straus SE, Hickie I, Sharpe MC, Dobbins JG, Komaroff A: **The chronic fatigue syndrome: a comprehensive approach to its definition and study. International Chronic Fatigue Syndrome Study Group.** *Ann Intern Med* 1994, **121:**953-959.
158. Smets EM, Garssen B, Bonke B, De Haes JC: **The Multidimensional Fatigue Inventory (MFI) psychometric qualities of an instrument to assess fatigue.** *J Psychosom Res* 1995, **39:**315-325.
159. Krupp LB, LaRocca NG, Muir-Nash J, Steinberg AD: **The fatigue severity scale. Application to patients with multiple sclerosis and systemic lupus erythematosus.** *Arch Neurol* 1989, **46:**1121-1123.
160. Chalder T, Berelowitz G, Pawlikowska T, Watts L, Wessely S, Wright D, Wallace EP: **Development of a fatigue scale.** *J Psychosom Res* 1993, **37:**147-153.
161. Reed JF, 3rd: **Analysis of two-treatment, two-period crossover trials in emergency medicine.** *Ann Emerg Med* 2004, **43:**54-58.
162. Senn S, Ezzet F: **Clinical cross-over trials in phase I.** *Stat Methods Med Res* 1999, **8:**263-278.
163. Senn S: **The AB/BA crossover: past, present and future?** *Stat Methods Med Res* 1994, **3:**303-324.
164. Cleare AJ, Blair D, Chambers S, Wessely S: **Urinary free cortisol in chronic fatigue syndrome.** *Am J Psychiatry* 2001, **158:**641-643.
165. Nakamoto M: **Responses of sympathetic nervous system to cold exposure in vibration syndrome subjects and age-matched healthy controls.** *Int Arch Occup Environ Health* 1990, **62:**177-181.

166. Sramek P, Simeckova M, Jansky L, Savlikova J, Vybiral S: **Human physiological responses to immersion into water of different temperatures.** *Eur J Appl Physiol* 2000, **81:**436-442.

167. Stewart JM: **Autonomic nervous system dysfunction in adolescents with postural orthostatic tachycardia syndrome and chronic fatigue syndrome is characterized by attenuated vagal baroreflex and potentiated sympathetic vasomotion.** *Pediatr Res* 2000, **48:**218-226.

168. Naschitz JE, Rosner I, Rozenbaum M, Gaitini L, Bistritzki I, Zuckerman E, Sabo E, Yeshurun D: **The capnography head-up tilt test for evaluation of chronic fatigue syndrome.** *Semin Arthritis Rheum* 2000, **30:**79-86.

169. Natelson BH, Intriligator R, Cherniack NS, Chandler HK, Stewart JM: **Hypocapnia is a biological marker for orthostatic intolerance in some patients with chronic fatigue syndrome.** *Dyn Med* 2007, **6:**2.

170. Wyller VB, Due R, Saul JP, Amlie JP, Thaulow E: **Usefulness of an abnormal cardiovascular response during low-grade head-up tilt-test for discriminating adolescents with chronic fatigue from healthy controls.** *Am J Cardiol* 2007, **99:**997-1001.

171. Schillings ML, Kalkman JS, van der Werf SP, van Engelen BG, Bleijenberg G, Zwarts MJ: **Diminished central activation during maximal voluntary contraction in chronic fatigue syndrome.** *Clin Neurophysiol* 2004, **115:**2518-2524.

172. Cohen J: *Statistical power analysis for the behavioral sciences.* 2nd edn. Hillsdale, NJ: Erlbaum; 1988.

173. Senn S: **Cross-over trials in Statistics in Medicine: the first '25' years.** *Stat Med* 2006, **25:**3430-3442.

174. Freeman PR: **The performance of the two-stage analysis of two-treatment, two-period crossover trials.** *Stat Med* 1989, **8:**1421-1432.

175. **Power Analysis and Sample Size software, NCSS** [http://www.ncss.com/pass.html]

176. Jacobson NS, Truax P: **Clinical significance: a statistical approach to defining meaningful change in psychotherapy research.** *J Consult Clin Psychol* 1991, **59:**12-19.

177. Reeves WC, Wagner D, Nisenbaum R, Jones JF, Gurbaxani B, Solomon L, Papanicolaou DA, Unger ER, Vernon SD, Heim C: **Chronic fatigue syndrome--a clinically empirical approach to its definition and study.** *BMC Med* 2005, **3:**19.

178. Vermeulen RC, Scholte HR: **Exploratory open label, randomized study of acetyl- and propionylcarnitine in chronic fatigue syndrome.** *Psychosom Med* 2004, **66:**276-282.

179. Castellani JW, Young AJ, Kain JE, Sawka MN: **Thermoregulatory responses to cold water at different times of day.** *J Appl Physiol* 1999, **87:**243-246.

180. Farquhar WB, Hunt BE, Taylor JA, Darling SE, Freeman R: **Blood volume and its relation to peak O_2 consumption and physical activity in patients with chronic fatigue.** *Am J Physiol Heart Circ Physiol* 2002, **282:**H66-71.
