# Supplementary material for: Possible use of repeated cold stress for reducing fatigue in chronic fatigue syndrome: a hypothesis
Source: Behav Brain Funct. 2007 Oct 24;3:55. doi: 10.1186/1744-9081-3-55 (PMC2164952; doi:10.1186/1744-9081-3-55)
Supplement: Additional file 3 — Limited preliminary evidence. The file name is Additional_File_3.pdf and it provides a detailed description of preliminary evidence that appears to support the hypothesis. The file contains its own list of references separate from the main text. [file 1744-9081-3-55-S3.pdf]

Additional file 3

Limited preliminary evidence

Described in this section is statistically insignificant evidence of the anti-fatigue effect of cold hydrotherapy. These are unpublished data, and all statements below are based on this limited evidence (except where references are provided).

A small percentage of population in Russia uses cold hydrotherapy on a regular basis (in all seasons) for example, cold affusions (i.e. pouring of 1-2 buckets full of cold water onto oneself) and cold showers, the practice that most likely derives from Russian folk medicine and there are virtually no reports on this practice in peer-reviewed literature [181]. It is worth mentioning that cold affusions were used by conventional medicine for the treatment of fever two centuries ago [182,183] and, in modern clinical practice, cold baths and cold water spraying/evaporation are used routinely in the management of heatstroke and severe hyperthermia [184,185]. The author found existing procedures to be rather stressful (and painful if water temperature is lower than 14°C [186,187]) and, therefore, he eventually modified cold showers to include a gradual 5-minute adaptation phase in order to make them more comfortable (which was in part prompted by valuable critical comments on cold hydrotherapy by Stephan Ladisch, M.D.).

The author has tested the effects of cold showers (15-20°C, 3-10 minutes) on himself for over 2 years almost daily. The first 7 months consisted of sudden cold showers and the rest of the experiment (up to now) consisted of adapted cold showers (twice a day, sometimes once a day for both periods; in the most recent months: every

other day mostly). The author found that twice daily cold showers (either sudden or adapted) can reduce a subjective sensation of fatigue caused by exercise or by an upper respiratory tract infection. The anti-fatigue effect seems to take place during a cold shower and can persist for several hours thereafter. The author interviewed two residents of Russia, whom he knows closely (healthy subjects, as is the case for the author) and who use cold affusions on a regular basis, about the effects of cold hydrotherapy. Both of them confirmed experiencing the reduction of exercise- and respiratory infection-related fatigue as a result of cold affusions. These people have been using cold affusions several times per month (or several times per day during a febrile illness): one person for approximately 17 years, the other for 19 years, with some extended periods of daily use (up to several months) in the past. They did not measure water temperature during cold affusions, and the temperature of tap cold water can vary widely throughout the year.

Multidimensional Fatigue Inventory scores [188] of the author over the last ~ 2 years are shown in Tables 3 and 4. The other two “participants” were reluctant to fill out the questionnaires apart from agreeing to answer a few questions. Neither the author nor the other two “participants” observed adverse somatic effects of cold hydrotherapy. With respect to neuropsychiatric effects, the author observed some usually undesirable effects as described below. In the experiment on himself, the author found that long-term use of twice daily adapted cold showers (16 months) may cause such symptoms as an irregular sleep pattern and/or low need for sleep, distractibility, and persistent agitated euphoria, all of which appear to be relieved if the frequency of cold showers is reduced 4-fold or more. These effects were mild but nevertheless resulted in reduced productivity at work. The other two participants did not report experiencing these symptoms as a result of cold

hydrotherapy, which may be due to the different frequency of cold water treatments as well as the different mode of administering cold stress (cold affusions vs. adapted cold showers). In addition, the author found that an adapted cold shower can shift forward (i.e. postpone) a sleep schedule if used at night, while the other two participants did not observe this effect with cold affusions. Further studies would be needed to assess the safety of daily moderate cold hydrotherapy in healthy subjects and in patients.

At present, the author continues testing the effects of adapted cold showers (20°C, 7-10 minutes total) on himself at a reduced frequency of once every 1-2 days. If sufficiently cold tap water is unavailable (in Richmond, Virginia, the coldest tap water during the summer is 28-30°C), the author uses cold baths (prepared by mixing ice with tepid water) also with gradual adaptation or, alternatively, tepid showers followed by running while wet in the atmosphere of cool air (10-15°C).

Table 3. The effects of daily cold hydrotherapy on Multidimensional Fatigue Inventory (MFI-20) scales of the author.

The data are presented in continuous chronological order[1] for the last ~2 years (left to right). Fatigue and other scales range from 4 (little or no fatigue or a most favorable reading of another type of scale) to 20 (severe fatigue or a worst reading of another type of scale). For example, if the value of Reduced Activity scale equals 4, this means full activity (high productivity), while the reading of 20 would roughly mean disability.

	Before regimen[2]	Throughout ~15 months of regimen	During the 16th month of regimen	No treatment for 6 weeks[3]	Throughout ~8 months of reduced regimen[4] (up to now)
**General Fatigue**	7	4	4	7	4
**Physical Fatigue**	5	4	4	5	4
**Reduced Activity**	6	5	13	6	5
**Reduced Motivation**	4	4	4	4	4
**Mental Fatigue[5]**	5	5	14	5	5

[1] MFI-20 questionnaires [188] for all columns except the last were filled out retrospectively.
[2] "Regimen" means cold showers twice a day (morning and afternoon); the first 7 months correspond to sudden cold showers, while the rest of the experiment involved adapted cold showers.
[3] No adverse withdrawal symptoms were observed, but the fatigue level gradually (over ~ 2 weeks) increased to pre-treatment levels.
[4] "Reduced regimen" means adapted cold showers once every 1 to 2 days.
[5] According to MFI-20, such symptoms as distractibility and racing thoughts may be rated as increased mental fatigue, even though there appears to be no perception of mental fatigue.

Table 4. The effects of daily cold hydrotherapy on Multidimensional Fatigue Inventory (MFI-20) scores of the author.

The data are presented in continuous chronological order[1] for the last ~2 years (left to right). Contents of MFI-20 are not shown because they are copyrighted by E.Smets, B.Garssen, and B.Bonke. A copy of MFI-20 can be obtained by contacting Dr. Ellen M.A. Smets by e-mail: e.m.smets@amc.uva.nl

item number	Before regimen[2]	Throughout ~15 months of regimen	During the 16th month of regimen	No treatment for 6 weeks	Throughout ~8 months of reduced regimen[3] (up to now)
**1**	2	1	1	2	1
**2**	1	1	1	1	1
**3**	2	2	1	2	2
**4**	1	1	1	1	1
**5**	2	1	1	2	1
**6**	2	1	4	2	1
**7**	1	1	4	1	1
**8**	1	1	1	1	1
**9**	1	1	1	1	1
**10**	1	1	4	1	1
**11**	1	1	3	1	1
**12**	1	1	1	1	1
**13**	2	2	3	2	2
**14**	2	1	1	2	1
**15**	1	1	1	1	1
**16**	2	1	1	2	1
**17**	1	1	4	1	1
**18**	1	1	1	1	1
**19**	1	1	4	1	1
**20**	1	1	1	1	1

[1] MFI-20 questionnaires [188] for all columns except the last were filled out retrospectively.
[2] "Regimen" means cold showers twice a day (morning and afternoon); the first 7 months correspond to sudden cold showers, while the rest of the experiment involved adapted cold showers.
[3] "Reduced regimen" means adapted cold showers once every 1 to 2 days.

References

181. Matveev VG: **Further comment on the precepts of Porfirii Ivanov.** *Med Sestra* 1990, **49:**59-60.
182. Forrester JM: **The origins and fate of James Currie's cold water treatment for fever.** *Med Hist* 2000, **44:**57-74.
183. Wright W: **Remarks on malignant fevers; and their cure by cold water and fresh air.** *Lond Med J* 1796, **7:**109-115.
184. Glazer JL: **Management of heatstroke and heat exhaustion.** *Am Fam Physician* 2005, **71:**2133-2140.
185. Wexler RK: **Evaluation and treatment of heat-related illnesses.** *Am Fam Physician* 2002, **65:**2307-2314.
186. Misasi S, Morin G, Kemler D, Olmstead PS, Pryzgocki K: **The effect of a toe cap and bias on perceived pain during cold water immersion.** *J Athl Train* 1995, **30:**49-52.
187. Casey KL, Minoshima S, Morrow TJ, Koeppe RA: **Comparison of human cerebral activation pattern during cutaneous warmth, heat pain, and deep cold pain.** *J Neurophysiol* 1996, **76:**571-581.
188. Smets EM, Garssen B, Bonke B, De Haes JC: **The Multidimensional Fatigue Inventory (MFI) psychometric qualities of an instrument to assess fatigue.** *J Psychosom Res* 1995, **39:**315-325.
